# Cali, Colombia, Key learning City C/Can 2025: City Cancer Challenge

**DOI:** 10.25100/cm.v48i2.3203

**Published:** 2017-06-30

**Authors:** Luis Eduardo Bravo, Olga Isabel Arboleda, Oscar Ramirez, Alexander Durán, Maria Cristina Lesmes, Melissa Rendler-García, Silvina Frech, Rolando Camacho, Susan Henshall

**Affiliations:** 1 Registro Poblacional de Cáncer de Cali, Departamento de Patología, Universidad del Valle, Cali, Colombia.; 2 Unión Internacional para el Control del Cáncer (UICC), Gerente del proyecto. Cali, Colombia.; 3 Fundación Pediatras OncoHEMAtólogos (Pohema). Cali, Colombia.; 4 Secretario de Salud Pública Municipal. Cali, Colombia.; Secretaria de Salud Departamental- Valle del Cauca, Cali, Colombia; 6 External consultants C/Can 2025 Special Advisor for City Implementation, Union for International Cancer Control. UICC, Geneva, Switzerland; 7 Union for International Cancer Control, (UICC). C/Can 2025 Programme Director. Geneva Switzerland

Cali (Colombia) will be the first city in the world to implement the initiative "C/Can 2025: City Cancer Challenge" ^1^ (http://www.uicc.org/what-we-do/convening/ccan-2025-city-cancer-challenge). With 2.4 million inhabitants, Cali is among the three largest cities of Colombia and a key urban focal point for the immigration of the country's rural population which represents one fifth of Colombia's 45.5 million inhabitants.

The C/Can 2025 initiative is led by the Union for International Cancer Control (UICC), the largest international cancer organization on a global scale, with more than 1,000 members in 162 countries. 

Through UICC's UN Economic and Social Council (ECOSOC) status, the organisation also works closely with key UN agencies including: the International Agency for Research on Cancer (IARC), United Nations Office for Drugs and Crime (UNODC), and International Atomic Energy Agency (IAEA).

Based on global best practices in cancer care, C/Can 2025 selected four main areas for implementation: 1) Basic cancer services; 2) management of cancer services; 3) quality of cancer care and 4) access to community and integrative care. For each of the areas, it is necessary to have a diagnosis of the initial situation, define processes, set goals, and assess results with a comprehensive measure of the overall impact of implementation ^1^ ^,^ ^2^.

C/Can 2025 urges the international community to work with cities with a population greater than one million to improve oncological care and treatment offered to their citizens through a network of committed partners, such as municipal leaders, governments, NGOs, UN agencies, national and international companies. It is important to work at city level because, today, 54% of the world's population lives in urban environments. This number is expected to rise to 66% by 2050. The global population living in medium-sized cities doubled between 1990 and 2014, and is projected to increase another 36% between 2014 and 2030, growing from 827 million to 1.1 billion. As the world continues to urbanize, sustainable development challenges will be increasingly concentrated in cities and, particularly in emerging cities experiencing rapid growth; therefore, cities are facing an increasing burden of cancer with an aging population in less healthy settings ^1^ ^,^ ^2^.

C/Can 2025: City Cancer Challenge is engaging all city stakeholders in the design, planning and implementation of cancer care solutions. Between now and 2025, the initiative will target over 200 cities to improve the health of at least 500 million people worldwide ^1^ ^,^ ^2^. To be part of the challenge, the cities must commit to its principles, and undertake a comprehensive needs assessment of cancer services to develop a strategic implementation to fill gaps in quality cancer care.

C/Can 2025 represents a paradigm shift in the way international organizations want to support national and municipal leaders to address the growing cancer burden. UICC and its partners are committed to work together with cities, as far as these invest in strategies and infrastructures for quality cancer care and treatment. It is expected that, by 2025, when the United Nations will measure the progress of the Objectives of Sustainable Development for Non-Communicable Diseases (NCDs) agreed upon by Member States in 2012, it will be possible to show how the C/Can2025 cities cure more patients with cancer.

The first three cities selected to implement C/Can 2025 were: Asunción (Paraguay), Cali (Colombia) and Yangon (Myanmar) ^1^ ^,^ ^2^. Cali was selected from hundreds of cities because of the progress made in cancer control and the great strength of the Cali Cancer Registry (RPCC, for its initials in English), which has operated continuously for the past 55 years. The information provided by the RPCC will serve as a baseline for C/Can 2025 and will provide important input to assess it in context with the National Plan for Cancer Control in Colombia. The UICC has a strong relationship with other local actors, the local civil society and the Colombian Ministry of Health. The other selected cities, Yangon and Asunción, will have limitations due to lack of information; one of their first objectives should be the creation of a population-based cancer registry.

Urbanization, growth and aging of the population require reorganizing health services and optimizing cancer care. As of August 2016, Cali has an offer of 163 cancer services, the majority of which are private (87.2%); the rest are public institutions (12.2%) and mixed (<1%) institutions ^3^. With these resources, Cali treats approximately 9,000 new patients per year, of which 55% come from out of the town ^4^. Cali's offer of oncological services is far from optimal. Survival observed at 5 years in children under 15 years of age is 52%, 20% below what can be reached in countries with greater economic development ^5^ ^,^ ^6^. Access to health insurance determines the prognosis, only one out of three poor uninsured children is alive five years after diagnosis ^7^. Those who have access to private insurance have a five-year survival rate of 84%, similar to what is expected in rich countries. In adults, the clinical outcome after diagnosis is also unfavorable. Survival to cancer is significantly lower compared to what is observed in rich countries ^3^ ^-^ ^5^. In the first five-year period of the 21st century, coinciding with the implementation of the new Colombian health system in the 1990s, survival increased for most malignancies compared to 1995-1999. This trend stagnated in the five-year period 2005-2009 ^5^ ^,^ ^6^.

C/Can 2025 is an opportunity to modernize Cali's network of public and private cancer services, and a challenge for regional and national academic institutions. Oncology must be incorporated into academic offerings to train technicians, professionals and specialists. It is necessary to evaluate the diagnostic services, the provision of service according to the infrastructure and the human resources available to put into practice the cancer treatment services, from diagnosis to palliative care.

Oncologic care is complex and has many potential risks. The decision-making process of motivated, qualified and multidisciplinary health professionals, committed to providing quality oncology diagnostics and curative and palliative treatments, is essential, as it can save lives and improve quality of life. This means having the right principles to ensure that all the right decisions are made. It is crucial to manage cancer care services so that they can function efficiently, responsibly, and sustainably, with high ethical standards and patient care at its core.

## References

[1] Adams C, Henshall S, Torode J, D'Cruz AK, Kumar HS, Aranda S (2017). C/Can 2025: City Cancer Challenge, a new initiative to improve cancer care in cities. Lancet Oncol.

[2] C/Can 2025 City Cancer Challenge (2017). Guiding Principles for Quality Cancer Treatment Services in Cities.

[3] Aguilera LJ, Murcia MEM (2016). Servicios Oncológicos de Colombia.

[4] Bravo LE, Collazos T, Collazos P, García LS, Correa P (2012). Trends of cancer incidence and mortality in Cali, Colombia 50 years experience. Colomb Med (Cali).

[5] Bravo LE, García LS, Collazos P, Aristizabal P, Ramirez O (2013). Descriptive epidemiology of childhood cancer in Cali Colombia 1977-2011. Colomb Med (Cali).

[6] Allemani C, Weir HK, Carreira H, Harewood R, Spika D, Wang XS (2015). Global surveillance of cancer survival 1995-2009: analysis of individual data for 25,676,887 patients from 279 population-based registries in 67 countries (CONCORD-2). Lancet.

[7] Ospina-Romero M, Portilla CA, Bravo LE, Ramirez O, VIGICANCER working group (2016). Caregivers' self-reported absence of social support networks is related to treatment abandonment in children with cancer. Pediatr Blood Cancer.

